# Sodium Bicarbonate Nebulized Therapy in Patients with Confirmed COVID-19

**DOI:** 10.34172/apb.2021.047

**Published:** 2020-10-14

**Authors:** Jalil Rashedi, Behroz Mahdavi Poor, Mohammad Asgharzadeh

**Affiliations:** ^1^Department of Laboratory Sciences, School of Paramedicine, Tabriz University of Medical Sciences, Tabriz, Iran.; ^2^Biotechnology Research Center, Tabriz University of Medical Sciences, Tabriz, Iran.

## Dear Editor,

A global outbreak of severe acute respiratory syndrome (SARS) caused by a new coronavirus (CoV-2) began in December 2019. It induced a novel identified sickness named coronavirus disease 2019 (COVID-19). The rapid emergence of COVID-19 along with the considerable illness and mortality of the disease made it a major public health issue. Due to the lack of effective treatments, an extensive attempt is under way to provide and examine antiviral drugs.

Coronaviruses are enveloped positive-stranded RNA viruses that replicate in the host cell cytoplasm. To release their nucleocapsid into the cell, they rely on the binding to the cellular receptors, angiotensin converting enzyme 2 (ACE2) for SARS-CoV-2, by major conformational changes of the virus spike glycoprotein (S) and entry into the cells by endocytosis.^1,2^ The created endosome, where a slightly acidic environment is required for optimum fusion of its membrane and virus envelope, followed by the stages of fusion, uncoating and release of the genome into the host cell cytoplasm.^3,4^ The protonation of critical residues on a viral envelope glycoprotein alters its conformation and exposes a hydrophobic “fusion peptide” domain to facilitate the fusion.^5^ Infection of SARS-CoV-2 may be prevented by treatment of cells with inhibitors of endosome acidification.

**Figure 1:**
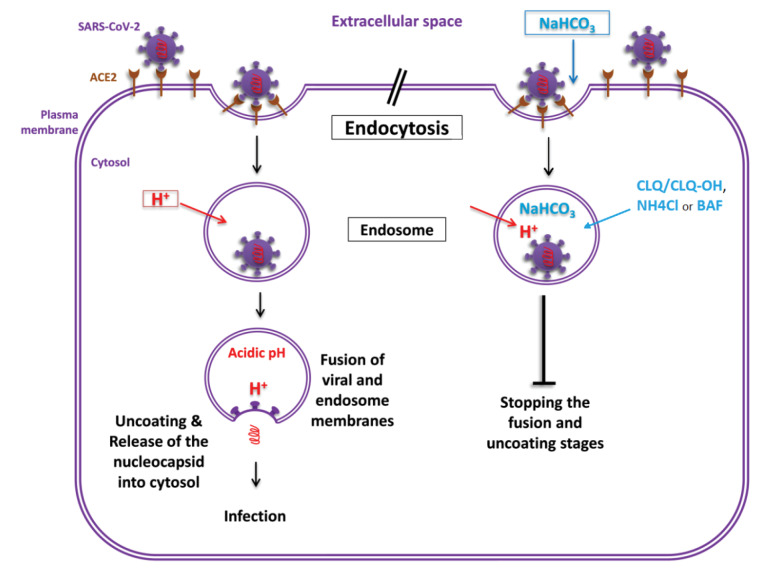
Assumed pathway of endocytosis of SARS-CoV-2 into the target cells with Sodium bicarbonate (right pathway) and without it (left one). Binding of the virus S-protein to ACE2 promotes internalization into endosomes, where the low pH environment triggers fusion activity in the S-protein. Inhibitors of acidification, such as NaHCO_3_, CLQ/CLQ-OH, NH_4_Cl or BAF can be used to dissect the pathway because they specifically block pH-dependent membrane fusion.

Sodium bicarbonate (NaHCO_3_), like endosomotropic weak bases ammonium chloride (NH_4_Cl), chloroquine (CLQ)/ hydroxychloroquine (CLQ-OH) as well as bafilomycin A (BAF), may raise the pH in vesicle/endosomes and would be expected to inhibit nucleocapsid release of a SARS-CoV-2 which required an acidic endosomal environment for uncoating (as shown in the Figure 1).^1,4,6,7^

Therefore, inhalation of the nebulized sodium bicarbonate solution (<5%) by the patients with confirmed COVID-19, several times during the day, probably able to stop the fusion and uncoating stages. In this case, the replication phase will also remain barren and eventually the respiratory infection will be controlled. Prospective controlled trials are needed to evaluate this method efficacy.

## Ethical Issues

Not applicable.

## Conflict of Interest

The authors declare that there is no conflict of interests.
